# COVID-19 vaccination and mortality among coronary care patients with severe acute respiratory infection in Bangladesh: a prospective study (2021–2024)

**DOI:** 10.1016/j.lansea.2026.100745

**Published:** 2026-03-02

**Authors:** Zubair Akhtar, Md Ariful Islam, Mohammad Abdul Aleem, Tanzir Ahmed Shuvo, Md Zakiul Hassan, Asadullah, Mustafizur Rahman, Mohammed Ziaur Rahman, Mohammad Enayet Hossain, Tahmina Shirin, Mahbubur Rahman, Manjur Hossain Khan Jony, Monalisa, Ferdous Rahman Sarker, Ahmed Nawsher Alam, Shah Niaz Md. Rubaid Anwar, Mahmudur Rahman, Awachana Jiamsakul, Aye M. Moa, Timothy C. Tan, Ole Fröbert, Fahmida Chowdhury, C Raina MacIntyre

**Affiliations:** a Infectious Diseases Division, icddr,b, Dhaka, Bangladesh; b Biosecurity Program, The Kirby Institute, Faculty of Medicine and Health, UNSW, Sydney, New South Wales, Australia; c School of Population Health, UNSW, Sydney, New South Wales, Australia; d Institute of Epidemiology, Disease Control and Research (IEDCR), Dhaka, Bangladesh; e EMPHNET, Bangladesh Office, Dhaka, Bangladesh; f Department of Cardiology, Blacktown Hospital, University of Western Sydney, 2148, Blacktown, NSW, Australia; g School of Medical Sciences, Faculty of Medicine and Health, UNSW, Sydney, NSW, Australia; h Department of Cardiology, Westmead Hospital, Sydney University, Westmead, NSW, Australia; i Örebro University, Faculty of Health, Department of Cardiology, Örebro, Sweden; j Department of Clinical Medicine, Faculty of Health, Aarhus University, Aarhus, Denmark; k Department of Clinical Pharmacology, Aarhus University Hospital, Arhus, Denmark; l Steno Diabetes Center Aarhus, Aarhus University Hospital, Aarhus, Denmark

**Keywords:** COVID-19 vaccination, AMI, All-cause mortality, CCU, Sentinel surveillance

## Abstract

**Background:**

COVID-19 increases cardiovascular risk, and vaccination reduces adverse outcomes and mortality. We analysed national hospital-based sentinel surveillance data from Bangladesh, and the aim of the study was to identify factors associated with all-cause mortality among patients with cardiovascular complications.

**Methods:**

We included patients from coronary care units in nine tertiary-hospitals between February 2021 and December 2024 with severe acute respiratory infections (SARI). Nasopharyngeal and oropharyngeal swabs were tested for SARS-CoV-2 and influenza viruses by multiplex rRT-PCR. Patients were followed up from hospital admission to 30 days post-discharge. Survival was assessed with Kaplan–Meier estimates stratified by vaccination status and compared using log-rank test. Risk factors for all-cause mortality were analysed using multivariable Cox proportional hazards regression, stratified by hospital type.

**Findings:**

We enrolled 396 patients (median age 60, IQR: 48–65 years), and 70.5% (279/396) were male. The Median follow-up time was 33 days (IQR: 32–34 days). There were 13.9% (55/396) deaths, 41.2% (163/396) had acute myocardial infarction (AMI) and 71.2% (286/396) were COVID-19 vaccinated patients. SARS-CoV-2 and influenza viruses were detected among 6.8% (27/396) and 4.8% (19/396) patients, respectively. At follow-up, the survival rate was 89.6% in COVID-19 vaccinated patients compared to 81.4% in unvaccinated patients (*P*-value = 0.041). AMI was associated with higher mortality [HR = 1.74, (95% CI: 1.01–3.02), *P*-value = 0.048] while COVID-19 vaccination was protective [HR = 0.55, (95% CI: 0.32–0.96), *P-*value = 0.037].

**Interpretation:**

COVID-19 vaccination was associated with reduced all-cause deaths among SARI patients with cardiovascular complications.

**Funding:**

Centres for Disease Control and Prevention (CDC), Atlanta, Georgia, USA (U01GH002259). ZA is supported by UNSW by a UIPA PhD scholarship.

**Research in context**

**Evidence before this study**

COVID-19 has been identified as a significant risk factor for cardiovascular events and mortality. While numerous studies have demonstrated the effectiveness of COVID-19 vaccination in reducing SARS-CoV-2-related deaths, limited research has explored its impact on cardiovascular outcomes and all-cause mortality, particularly in low-resource settings such as Bangladesh. Our search of PubMED for peer-reviewed articles from inception to October 31, 2025, using the terms covid^∗^ AND vaccin^∗^ AND effectiv^∗^ AND coronary care unit OR AMI AND mortality yielded twelve studies. To our knowledge, there is no evidence on the effectiveness of COVID-19 vaccines in reducing mortality among coronary care patients.

**Added value of this study**

This study assesses the link between COVID-19 vaccination and all-cause mortality in patients with cardiovascular issues in Bangladesh, using data from a comprehensive national hospital-based surveillance system. It shows a 45% reduction in all-cause mortality among vaccinated individuals, emphasising the cardio-protective advantages of vaccination in a real-world, resource-limited setting.

**Implications of all the available evidence**

The findings highlight the importance of COVID-19 vaccination in reducing mortality among high-risk cardiovascular patients. They endorse global vaccination programmes and suggest that wide vaccine coverage could lower the cardiovascular burden, especially in regions with limited healthcare infrastructure. This is especially important as COVID-19 vaccination rates are falling globally. Further research is necessary to investigate specific cardiovascular outcomes and to optimise vaccine strategies for vulnerable groups.

## Introduction

COVID-19 is an independent risk factor for cardiovascular events, particularly ischaemic stroke and acute myocardial infarction (AMI), both during and after acute SARS-CoV-2 infection^1^ and has been associated with increased mortality.^2^ As the pandemic progressed, numerous observational studies reported impairment in cardiac function,^2^, ^3^, ^4^ and sudden cardiac death.^5^ The cardiovascular mortality rate associated with SARS-CoV-2 infection has been reported to be between 6.7%^6^ and 73%^7^ among hospitalised patients with cardiovascular disease who developed COVID-19. Early reports from Bangladesh in 2020 documented a 14% mortality rate among patients hospitalised with ST-segment elevation myocardial infarction or non-ST-segment elevation myocardial infarction and concurrent COVID-19.^8^

COVID-19 is vaccine-preventable, and vaccination not only prevents infection but also reduces associated cardiovascular risk.^9^^,^^10^ Vaccine effectiveness (VE), particularly against death, has been a primary focus of research since the onset of the pandemic, as highlighted by the WHO^11^ and remains an ongoing area of research.^12^ A study conducted using the national US healthcare system showed that the COVID-19 VE in preventing SARS-CoV-2-related deaths was 86% (95% CI: 82%–89%).^13^ During 2023–2024 in the United States, 12% of annual COVID-19-associated in-hospital deaths were averted by COVID-19 vaccination.^14^ Although many studies have evaluated COVID-19 VE with regard to COVID-19-associated deaths,^15^ few studies have examined the benefits of the COVID-19 vaccines with regard to cardiovascular outcomes and all-cause deaths.

In Bangladesh, COVID-19 vaccines became available from January 2021, and mass vaccination was rolled out from February 2021.^16^ Although some evidence suggests a cardiovascular burden from COVID-19 in Bangladesh,^8^ data on the association between cardiovascular outcomes, all-cause mortality and COVID-19 vaccination remain limited in Bangladesh. This study used data from the national Hospital-based Influenza Surveillance (HBIS) system, which was collected from nine tertiary hospitals across Bangladesh, following the rollout of mass COVID-19 vaccination. We assessed determinants of all-cause mortality in patients with cardiovascular complications, with particular attention to COVID-19 vaccination status.

## Methods

This was a retrospective observational analysis, in which all-cause mortality was the primary outcome variable and COVID-19 vaccination the exposure of interest. The patients were recruited from HBIS in Bangladesh. This surveillance, initiated in 2007 by the National Influenza Centre, was conducted in nine tertiary hospitals across Bangladesh. The hospitals included seven public and two private institutions. This was a collaborative effort between the Institute of Epidemiology, Disease Control and Research (IEDCR) of the Government of Bangladesh, the icddr,b, and the U.S. Centres for Disease Control and Prevention (US CDC).^17^ The surveillance system was expanded to include SARS-CoV-2 detection as part of the COVID-19 pandemic response, starting in March 2020, in accordance with WHO recommendations.^17^^,^^18^ Even at the peak of the COVID-19 pandemic, surveillance operations were conducted six days a week (excluding Fridays) in inpatient departments of medicine and pediatrics, coronary care units (CCU), and specialised isolation wards that were established during the pandemic.^17^ This study presents findings on patients with severe acute respiratory infections (SARI) admitted to CCU for any cardiac condition in Bangladesh from February 2021 onwards, following the mass rollout of the COVID-19 vaccination, to December 2024.^16^

Each day from 8:30 am to 5:00 pm, study staff and physicians screened CCU inpatients admitted that day or after hours on the previous workday to identify eligible SARI cases, defined as a subjective fever or measured body temperature of ≥38 °C and cough onset within 10 days requiring hospitalisation. This case definition was adopted from the WHO^19^ and has been used in HBIS since May 2016.^17^ This study was conducted within an ongoing sentinel surveillance system. No sample size was calculated, and all participants meeting the SARI case definition who were admitted to the CCU, and who provided informed consent were included. No exclusion criteria were applied.

Following informed consent, the surveillance physicians conducted physical examinations of eligible patients and collected data using a standardised surveillance report form, which included questions on demographic information, vaccination status for influenza and COVID-19 (with at least one dose received). Detailed information on data collection was described in our earlier published article.^17^ All recruited patients with SARI had nasopharyngeal and oropharyngeal swabs, which were tested for SARS-CoV-2 and influenza virus types, subtypes/lineages by multiplex rRT-PCR. We recorded in-hospital all-cause mortality and at 30-days post-discharge. For patients who were discharged, study staff contacted patients or their family members on day 30 (+2) post-discharge to assess post-hospital status. Any deaths that occurred during the follow-up period were documented, including the date and place of death. It is important to note that admission to the CCU was determined according to local hospital protocols and guided by clinical presentation, medical history, and available diagnostic markers. Detailed indications for CCU admission were not systematically recorded within the surveillance system.

### Data analyses

We performed descriptive analyses to summarise covariate frequencies, including sociodemographic characteristics, year of admission, hospital type, medical and clinical history, diagnoses, COVID-19 and seasonal influenza vaccination status at admission, and viral test results. Data on race and ethnicity were not collected because these parameters are largely homogeneous in Bangladesh. Given the uncertainty regarding whether the missing-at-random assumption is satisfied, we have reported missing data as unknown in the tables. Risk time for all-cause mortality, expressed as 100 person-years (100PYS), was calculated from the date of hospital admission until death. Patients alive at follow-up were censored on 30 days post-discharge, based on their final follow-up status. No patient transfers or losses to follow-up were recorded. Survival time in days was analysed and displayed using Kaplan–Meier curves, stratified by COVID-19 vaccination status. A log-rank test was performed to assess differences in survival distributions between vaccinated and unvaccinated patients. Risk factors for all-cause mortality were analysed using stratified Cox proportional hazards regression with hospital of admission as the stratification variable. Stratification was performed to account for potential differences in baseline mortality risk between hospital settings, thus controlling for unmeasured differences in care quality or patient characteristics across hospital types. Regression models were fitted using a backward stepwise selection process. Patients with at least one day of follow-up were included in the regression analysis. Covariates with a *P-*value <0.10 in the univariate analysis were entered into the multivariate model. The covariates included in the final multivariate model were sex, dyspnoea, and AMI. Regression analysis based on viral strain could not be performed as the number of patients with individual strain identification was fewer than 20 per strain, which would preclude meaningful stratified analyses. A *P-*value <0.05 was considered statistically significant. Data management and statistical analyses were performed using Stata version 18.5 (StataCorp LP, College Station, TX, USA).

### Ethics approval and consent to participate

The study was approved by the icddr,b Institutional Review Board before enrolling participants (protocol # 2007-002) with latest annual approval on 12 May 2025. The US CDC's Human Research Protection Office approved a continuing reliance on the icddr,b IRB. Informed written consent to participate in the study was obtained.

### Role of funding source

The funders had no involvement in this work.

## Results

### Demographic characteristics, clinical characteristics and laboratory detections

**Fig. 1:**
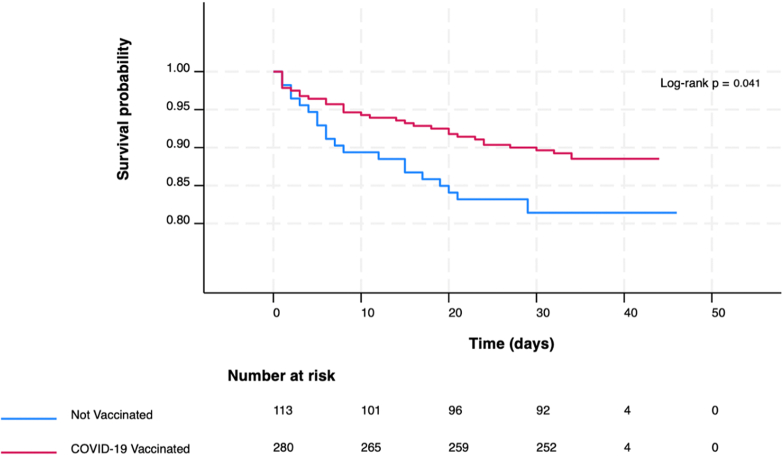
**Kaplan–Meier curve for all-cause mortality among COVID-19 vaccinated and unvaccinated patients admitted in coronary care units (CCU) in Bangladesh during February 2021–December 2024**.

We enrolled 396 patients with a median age of 60 years (Interquartile range, IQR: 48–65 years), and 70.5% (279/396) were male. The median follow-up time was 33 days (IQR: 32–34 days). There were 55 (13.9%, 55/396) deaths, with three patients dying on the day of hospital admission. Most patients (35.9%, 142/396) were enrolled in 2022, with the majority (55.8%, 221/396) from public hospitals. The characteristics of the study population are shown in Table 1. Dyspnoea was present in 82.6% (327/396) of patients and 90.9% (50/55) of the patients who died. Oxygen was required at admission in 72.2% (286/396) of patients and in 89.1% (49/55) of those who died. At hospital admission, AMI was diagnosed in 41.2% (163/396) and in 56.4% (31/55) of those who died. At enrolment, 71.2% (282/396) had received at least one dose of COVID-19 vaccination, and 28.8% (114/396) had not. Only one patient had received a seasonal influenza vaccination. SARS-CoV-2 was detected in 6.8% (27/396) of patients, and in 7.3% (4/55) of those who died. Influenza A/H3N2 was detected in 4.8% (19/396) and in 3.6% (2/55) of those who died. Fig. 1 shows the Kaplan–Meier survival curves for different COVID-19 vaccination groups. At 30-days, the survival probability was 89.6% for vaccinated patients and 81.4% for unvaccinated patients (p-log rank = 0.041). A total of 393 patients with at least one day of follow-up were included in the regression analysis. The total follow-up duration across all patients was 33.27 PYS. No co-infections were recorded between influenza viruses and SARS-CoV-2.

**Table 1:** Demographic and clinical characteristics of eligible patients admitted in coronary care units (CCU) in Bangladesh during February 2021–December 2024.

Characteristics	Total (%) N = 396, 100%	Deaths (%) N = 55, 13.9%
**Age, Median (IQR), years**	60 (48–65)	60 (50–65)
≤40 years	62 (15.7)	4 (7.3)
>40 years	334 (84.3)	51 (92.7)
**Sex**		
Male	279 (70.5)	46 (83.6)
Female	117 (29.6)	9 (16.4)
**Residence location**		
Rural	351 (88.6)	53 (96.4)
Urban	45 (11.4)	2 (3.6)
**Calendar Year of Enrolment**		
2021	101 (25.5)	19 (35.6)
2022	142 (35.9)	18 (32.7)
2023	90 (22.7)	13 (23.6)
2024	63 (15.9)	5 (9.1)
**Type of Hospital**		
Private	175 (44.2)	33 (60.0)
Public	221 (55.8)	22 (40.0)
**Smoking history**		
Never	171 (43.2)	17 (30.9)
Occasionally	118 (29.8)	22 (40.0)
Regularly	107 (27.0)	16 (29.1)
**Medical History**		
Asthma		
No	360 (90.9)	53 (96.4)
Yes	36 (9.1)	2 (3.6)
Chronic obstructive pulmonary disease		
No	353 (89.1)	48 (87.3)
Yes	43 (10.9)	7 (12.7)
Diabetes mellitus		
No	323 (81.6)	41 (74.6)
Yes	73 (18.4)	14 (25.5)
Hypertension		
No	266 (67.2)	37 (67.3)
Yes	130 (32.8)	18 (32.7)
Ischaemic heart disease		
No	294 (74.2)	38 (69.1)
Yes	102 (25.8)	17 (30.9)
**Clinical features**		
Chest pain		
No	273 (68.9)	34 (61.8)
Yes	123 (31.1)	21 (38.2)
Pneumonia		
No	117 (29.6)	18 (32.7)
Yes	89 (22.5)	14 (43.8)
Unknown	190 (48.0)	23 (41.8)
Dyspnoea		
No	69 (17.4)	5 (9.1)
Yes	327 (82.6)	50 (90.9)
Oxygen received at admission		
No	110 (27.8)	6 (10.9)
Yes	286 (72.2)	49 (89.1)
**Clinical diagnosis**		
Acute myocardial infarction		
No	233 (58.8)	24 (43.6)
Yes	163 (41.2)	31 (56.4)
Stable coronary artery disease		
No	372 (93.9)	53 (96.4)
Yes	24 (6.1)	2 (3.6)
Heart failure		
No	303 (76.5)	36 (65.5)
Yes	93 (23.5)	19 (34.6)
Arrhythmia		
No	387 (97.7)	54 (98.2)
Yes	9 (2.3)	1 (1.8)
**Vaccination status at admission**		
COVID-19		
No	114 (28.8)	22 (40.0)
Yes	282 (71.2)	33 (60.0)
Influenza		
No	395 (99.8)	55 (100.0)
Yes	1 (0.3)	0
**Viral infection**		
SARS-CoV-2		
No	368 (93.2)	51 (92.7)
Yes	27 (6.8)	4 (7.3)
Influenza		
No	359 (90.7)	52 (94.6)
A/H3N2	19 (4.8)	2 (3.6)
A/H1N1 (pdm09)	14 (3.5)	0
B (Victoria)	3 (0.8)	1 (1.8)
Unknown	1 (0.3)	0

### All-cause mortality

In multivariate analysis (Table 2) dyspnoea was associated with higher mortality [Hazard ratio (HR) = 2.59, (95% confidence interval, CI: 1.02–6.55), *P-*value = 0.045], as was AMI [HR = 1.74, (95% CI: 1.01–3.02), *P-*value = 0.048]. Factors associated with reduced mortality were female sex [HR = 0.43, (95% CI: 0.21–0.88), *P-*value = 0.021] and COVID-19 vaccination [HR = 0.55, (95% CI: 0.32–0.96), *P-*value = 0.037].

**Table 2:** Risk factors for all-cause mortality among patients admitted in coronary care units (CCU) in Bangladesh during February 2021–December 2024.

	Total patients (N = 393)^a^	Follow-up years (Total = 33.27 person-years, PYS)	No. of deaths (N = 52)^a^	Mortality-rates per 100 PYS (156/100 PYS)	Univariate HR (95% CI)	*P-*value	Multivariate HR (95% CI)	*P-*value
**Calendar year of hospitalization**^b^						0.256		
2021	101	8.13	19	234	Ref.			
2022	140	12.00	16	133	0.60 (0.31–1.17)	0.134		
2023	89	7.60	12	158	0.73 (0.35–1.51)	0.401		
2024	63	5.55	5	90	0.42 (0.15–1.11)	0.081		
**Age group**								
≤40 years	62	5.51	4	73	Ref.			
>40 years	331	27.76	48	173	2.44 (0.88–6.78)	0.086		
**Sex**								
Male	276	22.99	43	187	Ref.		Ref.	
Female	117	10.28	9	88	0.44 (0.22–0.91)	0.027	**0.43 (0.21–0.88)**	**0.021**
**Residence location**								
Urban	45	3.99	2	50	Ref.			
Rural	348	29.3	50	170	2.84 (0.69–11.78)	0.150		
**Smoking history**^b^						0.115		
Never	170	14.76	16	108	Ref.			
Occasionally	118	9.82	22	224	1.99 (1.03–3.77)	0.038		
Regularly	105	8.69	14	161	1.54 (0.75–3.15)	0.241		
**Medical history**								
Asthma								
No	357	30.11	50	166	Ref.			
Yes	36	3.16	2	63	0.48 (0.11–1.98)	0.308		
Chronic obstructive pulmonary disease								
No	350	29.68	45	152	Ref.			
Yes	43	3.59	7	199	1.06 (0.47–2.38)	0.885		
Diabetes mellitus								
No	320	27.38	38	139	Ref.			
Yes	73	5.89	14	238	1.61 (0.87–2.97)	0.131		
Hypertension								
No	263	22.15	34	154	Ref.			
Yes	130	11.12	18	162	1.18 (0.66–2.09)	0.582		
Ischemic heart disease								
No	292	24.78	36	145	Ref.			
Yes	101	8.49	16	189	1.30 (0.72–2.34)	0.386		
**Clinical features**								
Chest pain								
No	271	23.24	32	138	Ref.			
Yes	122	10.03	20	199	1.18 (0.66–2.11)	0.576		
Pneumonia^b^								
No	117	9.71	18	185	Ref.	0.994		
Yes	89	7.49	14	187	1.02 (0.51–2.04)	0.963		
Unknown	187	16.07	20	125	0.97 (0.47–2.02)	0.943		
Dyspnea								
No	69	6.00	5	83	Ref.		Ref.	
Yes	324	27.27	47	172	2.30 (0.91–5.81)	0.078	**2.59 (1.02–6.55)**	**0.045**
Oxygen received at admission								
No	110	9.78	6	61	Ref.			
Yes	283	23.49	46	196	2.64 (1.10–6.35)	0.030		
**Clinical diagnosis**								
Acute myocardial infarction								
No	232	20.20	23	114	Ref.		Ref.	
Yes	161	13.07	29	222	1.84 (1.06–3.18)	0.029	**1.74 (1.01–3.02)**	**0.048**
Stable coronary artery disease								
No	369	31.12	50	161	Ref.			
Yes	24	2.15	2	93	0.55 (0.13–2.28)	0.413		
Heart failure								
No	302	25.84	35	136	Ref.			
Yes	91	7.43	17	229	1.44 (0.79–2.60)	0.231		
Arrythmia								
No	384	32.49	51	157	Ref.			
Yes	9	0.78	1	128	0.82 (0.11–5.92)	0.842		
**Vaccination at hospitalization**								
COVID-19								
No	113	9.14	21	230	Ref.			
Yes	280	24.13	31	129	0.59 (0.34–1.02)	0.059	**0.55 (0.32–0.96)**	**0.037**
**Viral infection**								
SARS-CoV-2								
No	366	30.96	49	158	Ref.			
Yes	26	2.23	3	135	0.90 (0.28–2.89)	0.860		
Influenza^b^								
No	356	30.02	49	163	Ref.	0.932		
A/H3N2	19	1.60	2	125	0.76 (0.18–3.12)	0.703		
A/H1N1 (pdm09)	14	1.30	0	0	–			
B (Victoria)	3	0.27	1	377	1.67 (0.23–12.29)	0.612		
Unknown	1	0.09	0	0	–	–		

The covariates included in the final multivariate model were sex, dyspnea, and AMI.

Bold fonts are statistically significant.

a Three deaths occurred on hospital admission day and were not considered in the Cox regression analysis.

b Global *P*-value for calendar year of hospitalization, smoking history, pneumonia, and Influenza were tested for heterogeneity.

## Discussion

**Fig. 2:**
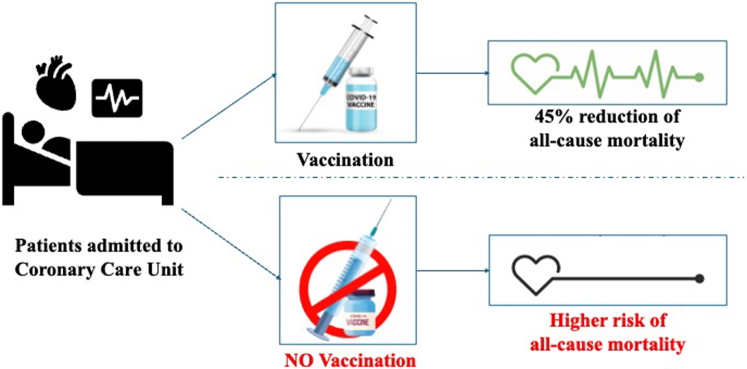
**Key study findings among SARI patients admitted in coronary care units (CCU) in Bangladesh during February 2021–December 2024**.

In this study, COVID-19 vaccination was associated with a 45% reduction of all-cause mortality among patients admitted to the CCU with severe acute respiratory infections in Bangladesh. At 30 days, survival was higher in COVID-19 vaccinated than in unvaccinated patients, and this survival advantage persisted throughout the study follow-up period. Our findings are consistent with those of the 2023 CVD-COVID-UK/COVID-IMPACT consortium, which analysed data from over 17 million individuals and reported that COVID-19 vaccination was associated with a reduced mortality risk. Specifically, vaccination was associated with a 64% lower mortality in all cases, a 61% reduction in those with cardiovascular disease, and a 71% lower mortality in those at high cardiovascular risk.^20^ Our estimates (45%), however, are lower compared to the UK study and other recent studies from Australia and Sweden, which demonstrate 58–90% efficacy in preventing mortality.^21^^,^^22^ However, ours was a special subset of people with both respiratory infection symptoms and cardiovascular disease. With limited studies reporting on the impact of COVID-19 vaccination on all-cause mortality^15^ our study adds to the existing body of knowledge (Fig. 2), especially in the context of a resource-limited setting like Bangladesh.

We found AMI to be significantly associated with an increased risk of mortality, with AMI diagnosed patients experiencing a 74% higher risk of all-cause mortality. Early reports from a population-based registry study conducted in Korea during the COVID-19 pandemic found that receiving two doses of the COVID-19 vaccine, spaced 28 days apart, was associated with a 52% lower risk of AMI.^10^ Another cohort study from the UK also indicated a 12–17% lower risk of AMI among vaccinated individuals under the age of 70 and a 25–26% lower risk in those over 70.^23^ Our findings are supported by contemporary evidence that the COVID-19 vaccine exhibits cardio-protective effects.^15^ Given the association between AMI and mortality, it is therefore not unreasonable to suggest that widespread vaccination may contribute to the alleviation of AMI-related deaths at the population level.

In our analysis, female sex was associated with reduced risk of all-cause mortality. This contrasts with earlier literature noting under-recognition of cardiovascular diseases in women and sex-specific differences in presentation^24^ that often lead to more conservative treatment.^25^ A recent meta-analysis found that women undergoing primary percutaneous coronary intervention for ST-elevation myocardial infarction experienced higher cardiac mortality than men.^26^ Similarly, other studies reported higher all-cause mortality among women compared to men.^27^ Although women generally experience higher short-term mortality after AMI, the lower mortality risk observed among women in this study contrasts with prior reports. This discrepancy may be partly explained by the short median follow-up period of 33 days, which differs from the longer follow-up durations in most AMI outcome studies. In addition, the absence of detailed markers of AMI severity may have contributed to residual confounding.

Strengths of the study include that data were obtained from a surveillance system operated continuously throughout the year, demonstrating resilience by remaining functional even during strict nationwide COVID-19 control measures.^28^ Secondly, surveillance data were uploaded in real time to local servers and centrally managed daily by a dedicated team, ensuring timely access and maintaining high standards of data quality. Lastly, biological samples were handled using strict protocols, and laboratory testing was conducted following standardised protocols established by the US CDC,^17^ which highlights the reliability and comparability of the results. Despite these strengths, we acknowledge several limitations of this study. Firstly, key clinical variables such as BMI, electrocardiograms, Troponin-I levels, malignancy, autoimmune diseases and use of immunosuppressive therapies were not collected, limiting adjustments for important effect modifiers or confounders. The analysis is subject to residual confounding from unmeasured variables inherent to the use of routinely collected surveillance data. Some potentially relevant factors were not captured, reflecting the surveillance system's design and scope rather than methodological oversight. Secondly, the clinical course of AMI was unavailable, which limited the ability to accurately document cardiac-related mortality based on the severity of AMI and its progression to heart failure and death. Detailed information on in-hospital management and acute clinical events, such as vasopressor use or cardiac arrest, was not available, precluding analyses of clinical endpoints beyond all-cause mortality. This limitation may have been further exacerbated by delayed patient presentations at the hospital during the pandemic period,^29^ potentially obscuring the cardiac-cause mortality. Third, information on COVID-19 vaccination dose number and timing was frequently incomplete, precluding analyses comparing partially vaccinated and fully vaccinated patients. Finally, as this study is based on ongoing sentinel surveillance, all participants who met the inclusion criteria and provided consent were included. However, the relatively small sample size may limit the generalizability of our findings. In part, this is because we selected a special subset of CVD patients, those presenting with symptoms of respiratory infection. Comparison of subjects without respiratory infection would have created more statistical power to study vaccination effects. Consequently, the findings should be interpreted as exploratory, providing real-world population-level estimates rather than causal inference.

In conclusion, COVID-19 vaccination was associated with a lower risk of all-cause mortality among patients admitted with SARI and underlying cardiovascular conditions in our study. Further studies should prospectively incorporate objective clinical indicators of AMI severity, including body mass index, electrocardiographic findings, and cardiac biomarkers such as troponin-I, to allow more precise adjustment for disease severity and to better characterise the association between COVID-19 vaccination and cardiovascular outcomes beyond all-cause mortality.

## Contributors

Study conceptualisation: ZA, MAA, MR, FC; Methodology: ZA, MAA, TAS, ARI, MAS, TSH, MR, M, AMM, TCT, OF, FC, CRM; Software: ZA, AJ; Investigation: ZA, ARI, MAA, TAS, TSH, MR, FC, MTR, MZR, MEH, MR2, MHKJ, M, FRS, ANA, MSNRA; Resources: ZA, FC; Formal analysis: ZA, AJ, AMM, FC; Data curation: ZA, Asadullah, AJ; Verifictaion: ZA, AJ, AMM; Original draft preparation: ZA; Supervision: TCT, OF, FC, CRM; Funding acquisition: ZA, TSH, MR, FC; Visualisation: ZA, AJ; Project administration: ZA, ARI, MAA, TAS, Asadullah, MSNRA, FC; Writing—review and editing: ZA, ARI, MAA, TAS, MTR, MZR, MEH, TSH, MR, MHKJ, M, FRS, ANA, MSNRA, AJ, AMM, TCT, OF, FC, CRM; All authors had access to the data and critically reviewed the manuscript for important intellectual content and approved the final version. ZA, FC and CRM had the final responsibility to submit for publication.

## Data sharing statement

Data generated during the study are subject to a data access policy of icddr,b and may be accessed from icddrb's research administration on reasonable request through the corresponding author.

## Declaration of interests

CRM reports Royalties for 2 books, Dark Winter and Vaccine Nation, along with honoraria for speaking at Options for the Prevention of Influenza conference from Sanofi, Pfizer, Moderna and Seqirus. OF reports consultancy fees from GSK and speaker's fees from Pfizer and Sanofi. TT reports consultancy fees from GSK and Bayer, as well as speaker's fees and support for meetings from GSK, Amgen, Novo Nordisk, Novartis, Boehringer Ingelheim, and Sanofi unrelated to the manuscript. He also participated in Advisory Boards from GSK, Sanofi Pasteur and Bayer. TCT is also on the Data and Safety Monitoring Board of PANDA and STRONGER trials. All these are unrelated to this manuscript. ZA, and other authors have nothing to declare.
